# Pre-screening and preventive quarantine likely explains the low SARS-CoV-2 prevalence among Norwegian conscripts

**DOI:** 10.1080/02813432.2021.1880101

**Published:** 2021-02-05

**Authors:** Arne J. Norheim, Einar K. Borud, Andreas Lind, Elin A. Fadum, Arne Taxt, Anneke Steens, Karoline Bragstad, Erling Rein, Espen Nakstad

**Affiliations:** a Norwegian Armed Forces Joint Medical Services, Sessvollmoen, Norway; b Department of Microbiology, Division of Laboratory Medicine, Oslo University Hospital-Ullevål, Oslo, Norway; c Norwegian Institute of Public Health, Oslo, Norway

**Keywords:** Covid-19, quarantine, military, motivating interview, PCR-test

## Abstract

**Objective:** We aim to discuss whether preventive quarantine can mitigate the spread of Covid-19 during the pandemic.

**Design:** We did a cross-sectional, observational study design in a mass-screening program in the enrolment to the Norwegian military during April 19–28th 2020 (COVID-NOR-MIL).

**Subjects:** 1170 presumptively healthy young Norwegian conscripts.

**Setting:** A structured interview encouraged the coming conscripts to a self-imposed preventive quarantine the last two weeks before enrolment.

**Main outcome measures:** All conscripts underwent a PCR-based test with nasopharyngeal swabs at the day of enrolment.

**Results:** Only two tested positive. The study discusses the predictive value of the RT-PCR test and the risk of false positive and false negative results, particularly when using the test in a low-prevalent cohort, even if the test properties of sensitivity and specificity is almost 100%. Further, the study discusses the challenge of whether a positive SARS-CoV-2 PCR-test represent viable and contagious virus or only viral remnants.

**Conclusion:** The adherence to self-imposed preventive quarantine is a challenge and is a subject to further research. Implications: We want to draw the attention to the potential value of a thorough pre-screening processes and self-imposed preventive quarantine to minimize the potential spread of SARS-Cov-2.

## Can pre-screening and preventive quarantine before enrolment lower the SARS-CoV-2 prevalence among Norwegian conscripts?

Physical distancing interventions have contributed to mitigate the coronavirus disease (Covid-19) pandemic. Mass-screening of 1170 presumptively healthy young Norwegian conscripts showed low PCR-based prevalence of SARS-CoV-2 in nasopharyngeal swabs where only two tested positive. This cross-sectional, observational are hypothesizing whether a self-imposed stay-home-policy and pre-screening before enrolment might contribute to the low prevalence.

Authorities in several countries and cities have imposed total lockdown to contain the Covid-19 outbreak [1]. Compliance to quarantine regimen highly depends on the possibility to perform necessary every-day activities and to the duration of the imposed quarantine measures [2] Even moderate restrictions are proven unfeasible in many societies in the long run [3].

Preventive quarantine is used to maintain an infection free environment among persons *without* symptoms or exposure to SARS-CoV-2 [4]. This was the motivation for the self-imposed preventive quarantine of Norwegian conscripts before enrolment to the military during the ongoing pandemic in Norway.

This short paper describes the RT-PCR-based SARS-CoV-2 prevalence among presumed healthy military conscripts, enrolled in the Norwegian army between April 19 and April 28, 2020.

### The corona virus disease project in the Norwegian military

The Corona Virus Disease Project in the Norwegian Military (COVID-NOR-MIL) is based on data obtained by extended systematic infection surveillance at enrolment to the Norwegian Armed Forces in April 2020.

A selection processes based on physical and mental skills, medical condition, and physical capacities is used to enrol about 9.000 of 60.000 eligible youth to the Norwegian Armed Forces each year. A total of 1802 youth (18–19 years of age) were selected for the April 2020 enrolment.

Among these, 624 conscripts applied for later enrolment or were removed from this specific enrolment for *non*-Covid-19 reasons. The remaining 1178 conscripts underwent a non-medical structured interview by telephone two weeks prior to entry, confirming that the conscript did not have any ongoing coronavirus disease they were aware of. This structured interview was performed by a non-medical military officer. The main purpose of this contact with the conscripts was to motivate them to enrol despite the Covid-19 pandemic, and to answer any questions related to the administrative parts of the enrolment to the military during the pandemic.

**Figure 1.**
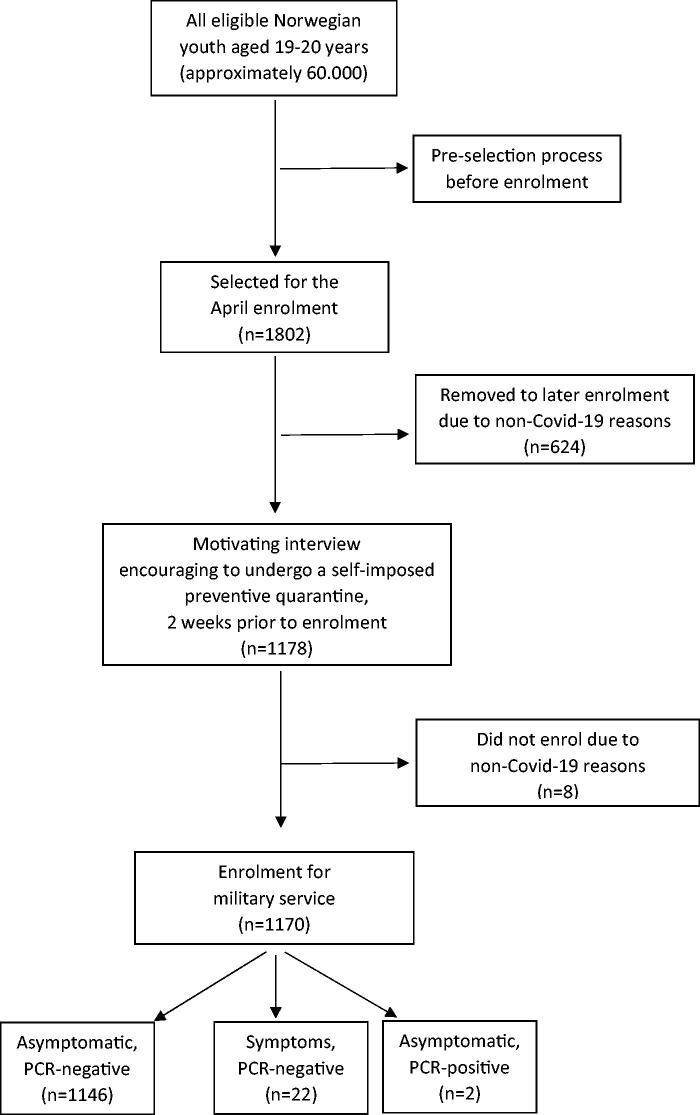
Flow chart of the participants included in the COVID-NOR-MIL study.

Additionally, the interviewer explained the importance of social distancing in the two last remaining weeks before enrolment, trying to maintain a SARS-CoV-2-free cohort entering the military. The conscripts were further strongly encouraged to undergo a self-imposed preventive quarantine [5]. This structured telephone interview might have motivated the conscripts, as only eight of the conscripts did not end up as conscripts (Figure 1).

The 1170 conscripts that did enrol for military service were, once again, asked whether they had a history of isolation or quarantine due to SARS-CoV-2 exposure. They did all confirm that they did not have any ongoing diagnosed or suspected coronavirus infection two weeks prior to enrolment. They gave their informed consent to participate and were thereafter included in this COVID-NOR-MIL mass-testing protocol. Two-thirds of the 1170 conscripts were men (799 men and 371 women) and the median age was 19 years and 7 months.

At the day of enrolment, all conscripts were asked in a questionnaire-pre-screening about any present symptoms which was reported from 22 conscripts. The reported symptoms among the 22 conscripts had the following percentage distribution: mild airways symptoms/cough (54%), sore throat (41%), headache (36%), diarrhoea (31%), shortness of breath (18%), fever (13%), reduced sense of taste or smelling (13%), dizziness (13%), and sore muscles (9%). All 22 conscripts with symptoms tested negative on RT-PCR for SARS-CoV-2 but were quarantined until they were free of any symptoms.

At the day of commencement, we obtained a nasopharyngeal and deep throat swab for RT-PCR detection of SARS CoV-2 RNA on all conscripts. The PCR tests were analysed at the Department of Microbiology at Oslo University Hospital using a Roche Cobas SARS-CoV-2 RNA test on the Cobas 6800 platform (Roche Diagnostics) with a the test sensitivity/specificity of 99% [6].

Only two conscripts (0.17%) had a positive RT-PCR test for SARS-CoV-2 at the day of enrolment. Both reported having had symptoms for more than two weeks before enrolment but reported no symptoms on the day of testing. Both these conscripts were confirmed IgG-positive in serological assays for SARS-CoV-2. We therefore assume these persons to be true positives.

At the time of enrolment, only 0.1% of the Norwegian population had been confirmed to have Covid-19 and the population point-prevalence was estimated to be approximately 1% by the Norwegian Institute of Public Health at enrolment [7]. The estimated reproductive/reproduction number (Re) for the period March 15–April 20^th^ is 0.67 (0.62–0.71) [8].

The proportion of positive tests among those who were tested had fallen over several weeks and was regarded as low at the time of the test-period. The age groups 10–19 and 20–29 years have the highest proportion of positive samples among those tested in the last two weeks, at 6.5% and 2.6%, respectively. The higher proportion of positive tests among the younger ones might, at least in part be explained by changed test criteria and more targeted testing in this group. The week before the COVID-NOR-MIL project, 1.5% of those who tested in the general population had a positive test [8].

## Discussion

Only 10% of Norwegian youth serve as military conscripts. Medical examinations and physical tests are the major constitutes of the pre-selection process. In addition, the coronavirus pandemic triggered additional individual telephone interviews with all conscripts to make it possible to withdraw from enrolment if any medical complaint or airway symptom was mentioned. The specific and unusual intervention for the conscripts was the strong encouragement to undergo preventive quarantine before enrolment.

This study among Norwegian military conscripts is a cross sectional ‘inspection’ of the covid-19 pandemic among healthy young adults and cannot be regarded as a true prevalence in the age-group, and particularly not for the general population. The timing for our study might perhaps be suboptimal as long as the pandemic situation in Norway during the enrolment period was characterized not only a low prevalence/incidence in the background population as mentioned, but also a low testing capacity and selective testing criteria. However, due to the relatively low point prevalence of Covid-19 among Norwegian military conscripts at enrolment (0.17%) we are hypothesizing whether this could be attributed to pre-screening and preventive measures before enrolment.

The predictive value of the RT-PCR test and the risk of false positive and false negative results can be questioned in this study. This is particularly important when using the test in a low-prevalent cohort, even if the test properties of sensitivity and specificity is almost 100%. Whether positive SARS-CoV-2 PCR represent viable and contagious virus or only viral remnants might vary depending on patients and stages of disease [9]. Due to the fact that our two conscripts also had positive serologic tests, we assume these two persons to be true positives.

The conscripts were asked about previous medical data including history of Cocid-19, isolation, and quarantine. However, they were not specifically asked about adherence to the self-imposed preventive quarantine introduced by the military officers. One explanation for this is that the self-imposed preventive quarantine was totally arranged by the military and was not a part of any medical procedure or intended medical measure. Consequently, we therefore do not have any reliable information whether the conscripts really complied to the encouraged self-imposed preventive quarantine.

The risk of spread of SARS-CoV-2 from asymptomatic carriers to the military may be reduced using preventive quarantine as described [10]. Preventive quarantine may have reduced the potential to become infected in the period before enrolment and thereby unintentionally introducing SARS-CoV-2 to the military during the incubation period or as asymptomatic carrier. The two conscripts who tested positive, might be fully recovered from the COVID-19 infection and therefore no longer infectious when they were tested.

After the summer, the epidemiological situation for Covid-19 in Norway has dramatically changed, and the increase is particularly prominent in young adults eligible for military service. Still the military has not introduced the test-protocol from COVID-NOR-MIL into mainstream enrolments after the study period April-June. Neither is there a standardized preventive quarantine or otherwise test-system upon enrolment, as for cross-border entry to Norway. Covid-19 has been found in many military campuses the last weeks, and one might speculate whether a regimen following COVD-NOR-MIL strategy would have lowered the prevalence of Covid-19 at enrolment into the Norwegian Armed Forces?

We want to draw the attention to the potential value of a thorough pre-screening processes and self-imposed preventive quarantine to minimize the potential spread of SARS-Cov-2. If more studies can conclude about an effect of preventive measures, there is a potential value within a variety of educational settings to mitigate the Covid-19 pandemic. This is important not only for the enrolment for military service, but also for other groups of persons or those that work without the possibility of strict physical and social distancing.
